# Genetic variants of DNA repair genes predict the survival of patients with esophageal squamous cell cancer receiving platinum-based adjuvant chemotherapy

**DOI:** 10.1186/s12967-016-0903-z

**Published:** 2016-05-31

**Authors:** Fei Zhou, Meiling Zhu, Mengyun Wang, Lixin Qiu, Lei Cheng, Ming Jia, Jiaqing Xiang, Qingyi Wei

**Affiliations:** Cancer Institute, Fudan University Shanghai Cancer Center, Shanghai, China; Department of Oncology, Fudan University Shanghai Medical College, Shanghai, China; Department of Oncology, Shanghai Jiaotong University Affiliated Shanghai First People’s Hospital, Shanghai, China; Department of Oncology, Xin Hua Hospital Affiliated to Shanghai Jiao Tong University, Shanghai, China; Department of Thoracic Surgery, Fudan University Shanghai Cancer Center, Shanghai, China; Duke Cancer Institute, Duke University Medical Center, 10 Bryn Searle Dr., Durham, NC 27710 USA

**Keywords:** Esophageal squamous cell cancer, Platinum-based adjuvant chemotherapy, Prognosis, DNA repair, Polymorphism, Biomarker

## Abstract

**Background:**

Adjuvant chemotherapy in patients with resected esophageal squamous cell cancer (ESCC) remains controversial for its uncertain role in improving overall survival (OS). Nucleotide excision repair (NER) removes DNA-adducts in tumor cells induced by the platinum-based chemotherapy and thus may modulate efficacy of the treatment. The present study evaluated if single nucleotide polymorphisms (SNPs) of NER genes were prognostic biomarkers in ESCC patients treated with platinum-based adjuvant chemotherapy (PAC).

**Methods:**

The analysis included 572 patients, for whom six SNPs of NER genes [i.e., *XPC* (rs1870134 and rs2228001), *ERCC2*/*XPD* rs238406 and *ERCC5*/*XPG* (rs2094258, rs2296147 and rs873601)] were detected with the TaqMan assay. Kaplan–Meier analyses and Cox proportional hazards models were used to evaluate their associations with disease free survival (DFS) and OS of these ESCC patients receiving PAC. Receiving operating characteristic curve analysis was used to evaluate the role of the risk genotypes in the DFS and OS.

**Results:**

We found that *ERCC5*/*XPG* rs2094258 and rs873601 and *ERCC2*/*XPD* rs238406 SNPs were independently associated with poorer DFS and OS of ESCC patients [*ERCC5*/*XPG* rs2094258: CT+TT vs. CC: adjusted hazards ratio (adjHR) = 1.68 and *P* = 0.012 for DFS; adjHR = 1.99 and *P* = 0.0001 for OS; *ERCC5*/*XPG* rs873601: GA+GG vs. AA: adjHR = 1.59 and *P* = 0.024 for DFS; adjHR = 1.91 and *P* = 0.0005 for OS; *ERCC2*/*XPD* rs238406: TT vs. GG+GT: adjHR = 1.43 and *P* = 0.020 for DFS; adjHR = 1.52 and *P* = 0.008 for OS]. These HRs increased as the number of risk genotypes increased in the combined analysis. The model combining the risk genotypes with clinical characteristics or the TNM stage system was better in predicting outcomes in ESCC patients with PAC.

**Conclusion:**

SNPs of *ERCC2*/*XPD* and *ERCC5*/*XPG* may independently and jointly predict survival of ESCC patients treated with PAC in this study population. Further validation in other study populations is warranted.

**Electronic supplementary material:**

The online version of this article (doi:10.1186/s12967-016-0903-z) contains supplementary material, which is available to authorized users.

## Background

Esophageal cancer (EC), more than 90 % of which are esophageal squamous cell carcinoma (ESCC), is the fourth leading cause of cancer-related deaths in China [1]. To date, surgery remains the standard treatment for resectable ESCC in China. But, for those patients who received an esophagectomy alone, their 5-year survival is still in a disappointing range of 15–40 % [2]. As a consequence, the surgery combined with adjuvant treatment has been employed to improve patients’ survival.

In recent years, neoadjuvant chemotherapy or neoadjuvant chemoradiotherapy has been widely introduced as a standard regimen by various guides. Unfortunately, the results of some major multicenter prospective randomized controlled trials (MPRCTs) were controversial. Besides, most of these trials were conducted in western countries, and more than 50 % of the patients included in these trials were diagnosed with an adenocarcinoma. Shapiro et al. [3] reported some overall survival (OS) benefits when neoadjuvant chemoradiotherapy was added to surgery, while others all reported opposite conclusions [4–6]. Furthermore, the only one MPRCT conducted in Hong Kong Chinese patients with ESCC reported a negative conclusion [7]. Of the four MPRCTs employing neoadjuvant chemotherapy, two reported benefits in OS [8, 9] but the other two did not [10, 11].

Adjuvant chemotherapy is not taken seriously in clinical practice, because it is not thought as effective as it should be, and it may have impaired patients’ functions as a result of esophagectomy and prolonged convalescence that hamper patients’ timely administration of adjuvant therapy [12]. To date, only three MPRCTs about the efficacy of platinum-based adjuvant chemotherapy (PAC) for EC have been published, among which two were conducted in Japanese ESCC patients. Although one did not yield the expected results [13], the other larger trial found an enhanced 5-year disease free survival (DFS) for patients with lymph node metastasis [14]. Traditionally, the anatomic and pathologic staging has been the most commonly used prognostic factors in EC patients, but it did not provide sufficient information for evaluating the efficacy of PAC, because it did not account for host factors, because genetic variants may interact with PAC and thus play a role in determining clinical response and prognosis of the patients.

In China, PAC is still the preference of many oncologists to treat EC patients [15]. Platinum compounds produce DNA adducts by reacting with DNA to form both intrastrand and interstrand cross-links in tumor cells, mainly with the N^7^ atom of guanine. These adducts result in a bulky distortion of the DNA helix, inhibit DNA replication, and eventually lead to cell death, if not repaired [16]. The amount of DNA adducts accumulated in tumor cells were correlated with the efficacy of platinum therapy and had an impact on clinical outcome of the patients [17].

Nucleotide excision repair (NER), which participates in two path ways of DNA repair, global genomic repair and transcription-coupled repair, plays an important role in detection and repair of DNA damage caused by UV, tobacco-related carcinogens and other carcinogenic chemicals [18, 19], including DNA adducts formed by platinum [20]. Several xeroderma pigmentosum (XP) group genes are involved in NER, including group C (*XPC*), group D (*ERCC2*/*XPD*) and group G (*ERCC5*/*XPG*) [21–24]. Several studies suggested that single nucleotide polymorphisms (SNPs) in these three genes might be responsible for the variation in DNA repair capacity, leading to individual variation in cancer susceptibility and treatment response [25–28]. However, few reported studies have studied the SNPs’ roles in the prognosis of EC patients treated with PAC.

Therefore, we hypothesize that potentially functional SNPs of the three NER genes may modulate prognosis of EC patients treated with PAC. In this study, we selected six well-studied potentially functional SNPs of the NER genes, including three from *ERCC5*/*XPG* (rs2094258 at 5′ near gene, rs22961475 at 5′ untranslated region (UTR) and rs873601 at 3′UTR), one from *ERCC2*/*XPD* (rs238406 at codon Arg156Arg) and two from *XPC* (rs1870134 at codon Val16Leu and rs2228001 at codon Lys939Gln) and evaluated their roles in survival of ethnic Han Chinese patients in eastern China.

## Methods

### Study population

The present study was done in a retrospective patient cohort in Fudan University Shanghai Cancer Center (Shanghai, China), and the research protocol was approved by the Institutional Ethics Review Board. Written informed consents were obtained from all patients before blood samples were obtained for genotype testing. Patients with perioperative mortality, defined as a death within 30 days of the operation or during the same hospitalization period, were excluded from the analysis. As a result, a cohort of 572 patients of ethnic Han Chinese in eastern China, who received an esophagectomy and had pathologically confirmed ESCC in the Department of Thoracic Surgery between March 2009 and December 2010, were included in the present study. Of these patients, additional 228 patients were excluded for the following reasons: 159 patients without undergoing postoperative chemotherapy for stage I disease, 35 patients without complete follow-up information, 7 patients for neoadjuvant chemotherapy and 27 patients for postoperative chemoradiotherapy. Therefore, the final analysis included 344 patients who completed four cycles of adjuvant chemotherapy (Oxaliplatin 135 mg/m^2^ d1 or cisplatin 40 mg/m^2^ d1–3 plus 5-Fu 750 mg/m^2^ d1–5).

Demographic and clinical information of the patients was abstracted from the medical records. All patients were staged according to the 7th edition of the American Joint Committee on Cancer staging system. Survival data were obtained through the follow-up in outpatient clinics or by telephone calls quarterly upto Oct. 31, 2014. The DFS was defined as the time interval between the date of surgical resection and the first confirmed detection of local recurrence or the appearance of new metastases. The OS duration of a patient was defined as the interval between surgical resection and the date of the latest follow-up or the death of the patients from any cause.

### SNP selection and genotyping

We selected six potentially functional SNPs from the NCBI dbSNP database (http://www.ncbi.nlm.nih.gov/) and the SNPinfo (http://snpinfo.niehs.nih.gov/). Genomic DNA was extracted from the buffy-coat fraction of the blood samples using the Qiagen Blood DNA Mini Kit (Qiagen Inc., Valencia, CA). All the six SNPs were genotyped using the Taqman real-time PCR method with a 7900 HT sequence detector system (Applied Biosystems, Foster City, CA). The primers used in genotyping for these SNPs are listed in Additional file 1: Table S1. To ensure high genotyping accuracy, strict quality control procedures were implemented, and four duplicated positive controls and four negative controls (no DNA) were used in each of 384-well plates. Approximately 5 % of the samples were repeatedly genotyped, and the results were 100 % concordant.

### Statistical methods

Cox proportional hazards regression analysis was used to evaluate the effect of genotypes and clinicopathological variables on patients’ DFS and OS, calculated as hazards ratios (HRs) with their corresponding 95 % confidence intervals (CIs). Kaplan–Meier analysis was used to present the visual effects of clinicopathological and genetic variables on the cumulative probability of DFS and OS. Receiver operating characteristic (ROC) analysis was used to compare sensitivity and specificity of predicting overall survival by the parameters. Statistical significance of the improvement in area under the receiver operator characteristic curve (AUC) after adding an explanatory factor was calculated by Delong’s test [29]. All reported *P* values were two-sided, and *P* < 0.05 was considered statistically significant. All analyses were performed using SAS software (version 9.2; SAS Institute, Cary, NC).

## Results

### Demographics and clinicopathological characteristics of ESCC patients and their associations with DFS and OS

The final analysis included 344 ESCC patients who received esophagectomy and PAC (Table 1) and had complete data on demographics, clinical characteristics, genotyping, DFS and OS. These patients aged between 37 and 77 years at the time of diagnosis with a mean of 58.43 years and a standard deviation of 8.03 years. More patients were men than women (85.8 vs. 14.2 %), with 33.7 % of stage II and 66.3 % of stage III diseases, among whom 81.7 and 18.3 % underwent radical operation through two-field and three-field lymphadenectomy, respectively. The median follow-up time was 36.13 months, during which 196 (57.0 %) patients died at the last follow-up. In multivariate analysis, three variables, i.e., TNM stage [adjusted hazards ratio (adjHR) = 1.55 and 95 % CI 1.13–2.12 for III vs. II], vessel invasion (adjHR = 1.44 and 95 % CI 1.07–1.94 for yes vs. no), and lymphadenectomy (adjHR = 1.42 and 95 % CI 1.03–1.97 for three fields vs. two fields), were significantly associated with DFS (*P* < 0.05). The two variables, TNM stage (adjHR = 1.49 and 95 % CI 1.06–2.08 for III vs. II) and vessel invasion (adjHR = 1.56 and 95 % CI 1.14–2.12 for yes vs. no) remained to be independent prognostic factors for OS (*P* < 0.05), but smoking (adjHR = 1.44 and 95 % CI 1.01–2.07 for yes vs. no) instead of lymphadenectomy became the third independent prognostic factor for OS.

**Table 1:** Associations of demographics and clinicopathological characteristics with DFS and OS of Chinese ESCC patients

Parameters	No. of patients	DFS					OS				
		Progression	Univariate analysis		Multivariate analysis*		Death	Univariate analysis		Multivariate analysis*	
		No. (%)	HR (95 % CI)	*P* value	HR (95 % CI)	*P* value	No. (%)	HR (95 % CI)	*P* value	HR (95 % CI)	*P* value
Age				0.287		0.295			0.123		0.087
<60	181	110 (60.8)	1.00		1.00		95 (52.5)	1.00		1.00	
≥60	163	110 (67.5)	1.15 (0.89–1.50)		1.15 (0.88–1.51)		101 (91.8)	1.25 (0.94–1.65)		1.28 (0.96–1.70)	
Sex				0.083		0.816			0.189		0.847
Male	295	194 (65.8)	1.00		1.00		173 (58.6)	1.00		1.00	
Female	49	26 (53.1)	0.69 (0.46–1.05)		0.82 (0.51–1.30)		23 (46.9)	0.72 (0.48–1.15)		0.95 (0.58–1.56)	
Smoking				0.080		0.250			0.035		0.046
Never	122	72 (59.0)	1.00		1.00		61 (50.0)	1.00		1.00	
Yes	222	148 (66.7)	1.28 (0.97–1.70)		1.22 (0.87–1.70)		135 (60.8)	1.39 (1.02–1.88)		1.44 (1.01–2.07)	
Drinking				0.192		0.970			0.302		0.855
No	171	103 (60.2)	1.00		1.00		92 (53.8)	1.00		1.00	
Yes	173	117 (67.6)	1.19 (0.92–1.56)		1.01 (0.75–1.36)		104 (60.1)	1.16 (0.88–1.54)		0.97 (0.71–1.33)	
Vessel invasion				0.0006		0.015			0.0002		0.005
No	243	142 (58.44)	1.00		1.00		122 (50.21)	1.00		1.00	
Yes	101	78 (72.23)	1.61 (1.22–2.13)		1.44 (1.07–1.94)		74 (73.27)	1.72 (1.29–2.30)		1.56 (1.14–2.12)	
Neural invasion				0.831		0.481			0.505		0.787
No	254	161 (63.39)	1.00		1.00		141 (55.51)	1.00		1.00	
Yes	90	59 (65.56)	1.03 (0.77–1.39)		0.90 (0.66–1.22)		55 (61.11)	1.11 (0.81–1.52)		0.96 (0.69–1.32)	
TNM stage				<0.0001		0.007			0.0003		0.021
II	116	58 (50.00)	1.00		1.00		52 (44.83)	1.00		1.00	
III	228	162 (71.05)	1.80 (1.33–2.43)		1.55 (1.13–2.12)		144 (63.16)	1.78 (1.29–2.44)		1.49 (1.06–2.08)	
Lymphadenectomy				0.003		0.035			0.006		0.066
Two fields	281	172 (61.21)	1.00		1.00		153 (54.45)	1.00		1.00	
Three fields	63	48 (76.19)	1.62 (1.18–2.24)		1.42 (1.03–1.97)		43 (68.25)	1.61 (1.14–2.25)		1.38 (0.98–1.95)	

* Adjusted for all parameters listed in Table 1

### Associations of selected SNPs with DFS and OS of ESCC patients

We assessed associations of six SNPs with DFS and OS of the 344 ESCC patients. In the multivariate analyses with adjustment for all the variables listed in Table 1, we found that DFS of the patients was significantly associated with *ERCC5*/*XPG* rs2094258 (CT+TT vs. CC: adjHR = 1.68, 95 % CI 1.23–2.31, and *P* = 0.012), rs873601 (GG+GA vs. AA: adjHR = 1.59, 95 % CI 1.06–2.37, and *P* = 0.024), and *ERCC2*/*XPD* rs238406 (TT vs. GG+GT: adjHR = 1.43, 95 % CI 1.06–1.93, and *P* = 0.020) (Table 2). Similarly, we also found in the multivariate analyses that OS of the patients was significantly associated with *ERCC5*/*XPG* rs2094258 (CT+TT vs. CC: adjHR = 1.99, 95 % CI 1.40–2.81, and *P* = 0.0001), rs873601 (GG+GA vs. AA: adjHR = 1.91, 95 % CI 1.21–2.99, and *P* = 0.0005), and *ERCC2*/*XPD* rs238406 (TT vs. GG+GT: adjHR = 1.52, 95 % CI 1.12–2.03, and *P* = 0.008) (Table 2).

**Table 2:** Associations between NER genetic variants and DFS and OS of Chinese ESCC patients

NER genotypes	No. of patients	DFS					OS				
		Progression	Univariate analysis		Multivariate analysis*		Death	Univariate analysis		Multivariate analysis*	
		No. (%)	HR (95 % CI)	*P* value	HR (95 % CI)	*P* value	No. (%)	HR (95 % CI)	*P* value	HR (95 % CI)	*P* value
*ERCC5* rs2094258											
CC	108	53 (49.1)	1.00		1.00		42 (38.9)	1.00		1.00	
CT	181	128 (70.7)	1.78 (1.29–2.46)	0.0004	1.70 (1.23–2.36)	0.001	118 (65.2)	2.06 (1.45–2.93)	<0.0001	1.98 (1.38–2.83)	0.0002
TT	55	39 (70.9)	1.72 (1.14–2.61)	0.010	1.63 (1.07–2.48)	0.021	36 (65.5)	2.07 (1.32–2.23)	0.001	2.02 (1.29–3.16)	0.002
CT+TT	236	167 (70.8)	1.76 (1.30–2.41)	0.0003	1.68 (1.23–2.31)	0.012	154 (65.3)	2.06 (1.46–2.90)	<0.0001	1.99 (1.40–2.81)	0.0001
Trend test				0.002		0.0007			0.0002		0.0005
CC+CT	289	181 (62.6)	1.00		1.00		160 (55.4)	1.00		1.00	
TT	55	39 (70.9)	1.19 (0.84–1.68)	0.331	1.16 (0.82–1.64)	0.415	36 (65.5)	1.28 (0.89–1.84)	0.178	1.29 (0.90–1.86)	0.171
*ERCC5* rs2296147											
TT	244	160 (65.6)	1.00		1.00		145 (59.4)	1.00		1.00	
TC	91	56 (61.5)	0.91 (0.67–1.23)	0.524	0.91 (0.67–1.24)	0.563	49 (53.9)	0.87 (0.63–1.21)	0.411	0.92 (0.66–1.29)	0.603
CC	9	4 (44.4)	0.52 (0.19–1.42)	0.201	0.49 (0.18–1.34)	0.165	2 (22.2)	0.26 (0.06–1.05)	0.058	0.27 (0.07–1.09)	0.066
TC+CC	100	60 (60.0)	0.86 (0.64–1.16)	0.333	0.87 (0.64–1.17)	0.345	51 (51.0)	0.80 (0.58–1.10)	0.172	0.84 (0.61–1.16)	0.289
Trend test				0.214		0.206			0.067		0.120
TT+CT	335	216 (64.5)	1.00		1.00		194 (57.9)	1.00		1.00	
CC	9	4 (4.4)	0.54 (0.20–1.45)	0.219	0.51 (0.19–1.37)	0.180	2 (22.2)	0.27 (0.07–1.08)	0.065	0.28 (0.07–1.12)	0.071
*ERCC5* rs873601											
AA	56	29 (51.8)	1.00				22 (39.3)				
GA	189	126 (66.7)	1.58 (1.05–2.36)	0.027	1.63 (1.08–2.47)	0.021	112 (59.3)	1.83 (1.16–2.89)	0.010	1.89 (1.19–3.02)	0.008
GG	99	65 (65.7)	1.56 (1.01–2.42)	0.047	1.51 (0.97–2.34)	0.069	62 (62.6)	1.99 (1.22–3.24)	0.006	1.93 (1.18–3.16)	0.009
GA+GG	288	191 (66.3)	1.57 (1.06–2.32)	0.024	1.59 (1.06–2.37)	0.024	174 (39.3)	1.89 (1.21–2.94)	0.005	1.91 (1.21–2.99)	0.0005
Trend test				0.084		0.146			0.011		0.021
AA+GA	245	155 (63.3)	1.00				134 (54.7)	1.00			
GG	99	65 (65.7)	1.09 (0.82–1.46)	0.536	1.04 (0.77–1.39)	0.803	62 (62.6)	1.23 (0.91–1.67)	0.168	1.18 (0.87–1.60)	0.297
*ERCC2* rs238406											
GG	102	58 (56.9)	1.00		1.00		50 (49.0)	1.00		1.00	
GT	158	101 (63.9)	1.16 (0.84–1.61)	0.361	1.15 (0.83–1.60)	0.403	87 (55.1)	1.21 (0.85–1.71)	0.256	1.23 (0.87–1.75)	0.247
TT	84	61 (72.6)	1.59 (1.11–2.28)	0.012	1.56 (1.08–2.24)	0.017	59 (70.2)	1.76 (1.21–2.57)	0.003	1.72 (1.18–2.52)	0.005
GT+TT	242	162 (66.9)	1.29 (0.96–1.75)	0.094	1.28 (0.94–1.73)	0.115	146 (60.3)	1.38 (1.00–1.91)	0.049	1.40 (1.01–1.93)	0.044
Trend test				0.013		0.019			0.004		0.005
GG+GT	260	159 (61.2)	1.00		1.00		137 (52.7)	1.00		1.00	
TT	84	61 (72.6)	1.45 (1.08–1.95)	0.014	1.43 (1.06–1.93)	0.020	59 (70.2)	1.57 (1.15–2.13)	0.004	1.52 (1.12–2.03)	0.008
*XPC* rs1870134											
GG	181	111 (61.3)					94 (51.9)	1.00		1.00	
GC	144	95 (66.0)	1.08 (0.82–1.41)	0.605	1.10 (0.83–1.46)	0.495	89 (61.8)	1.21(0.91–1.62)	0.192	1.25 (0.93–1.68)	0.137
CC	18	14 (73.7)	1.67 (0.96–2.92)	0.070	1.41 (0.80–2.48)	0.235	13 (68.4)	1.77 (0.99–3.17)	0.053	1.52 (0.84–2.75)	0.164
GC+CC	163	109 (66.9)	1.13 (0.87–1.47)	0.374	1.13 (0.87–1.48)	0.356	102 (62.6)	1.26 (0.96–1.67)	0.101	1.28 (0.96–1.70)	0.088
Trend test				0.171		0.242			0.045		0.066
GG+GC	325	206 (63.4)	1.00		1.00		183 (56.3)	1.00		1.00	
CC	19	14 (73.7)	1.62 (0.94–2.79)	0.081	1.35 (0.78–2.35)	0.285	13 (68.4)	1.62 (0.92–2.85)	0.093	1.38 (0.78–2.45)	0.274
*XPC* rs2228001											
TT	139	87 (62.6)	1.00		1.00		77 (55.4)	1.00		1.00	
TG	155	101 (65.2)	1.10 (0.82–1.46)	0.537	1.19 (0.89–1.59)	0.250	89 (57.4)	1.05 (0.77–1.42)	0.758	1.15 (0.84–1.57)	0.377
GG	50	32 (64.0)	1.18 (0.79–1.77)	0.418	1.29 (0.85–1.96)	0.238	30 (60.0)	1.25 (0.82–1.91)	0.295	1.36 (0.88–2.12)	0.171
TG+GG	205	133 (64.9)	1.12 (0.85–1.46)	0.432	1.21 (0.92–1.59)	0.181	119 (58.0)	1.09 (0.82–1.46)	0.539	1.19 (0.89–1.60)	0.241
Trend test				0.713		0.593			0.386		0.282
TT+TG	294	188 (64.0)	1.00		1.00		166 (56.5)	1.00		1.00	
GG	50	32 (64.0)	0.89 (0.61–1.29)	0.531	1.17 (0.80–1.73)	0.418	30 (60.0)	1.22 (0.83–1.80)	0.313	1.26 (0.84–1.89)	0.263
NRG**											
0	37	18 (48.7)	1.00		1.00		12 (32.4)	1.00		1.00	
1	63	30 (47.6)	0.97 (0.54–1.17)	0.789	1.09 (0.60–1.98)	0.920	25 (39.7)	1.22 (0.61–1.42)	0.578	1.38 (0.69–2.79)	0.365
2	187	127 (67.9)	1.32 (1.03–1.69)	0.030	1.32 (1.03–1.71)	0.021	115 (61.5)	1.53 (1.14–2.06)	0.005	1.55 (1.15–2.10)	0.005
3	57	45 (79.0)	1.34 (1.12–1.61)	0.002	1.34 (1.11–1.61)	0.0009	44 (77.2)	1.51 (1.22–1.87)	<0.0002	1.51 (1.22–1.88)	0.0002
Trend test				<0.0001		<0.0001			<0.0001		<0.0001
0–1	100	48 (48.0)	1.00		1.00		37 (37.0)	1.00		1.00	
2–3	244	172 (70.5)	1.69 (1.22–2.35)	0.002	1.67 (1.20–2.34)	0.003	159 (65.2)	1.84 (1.32–2.58)	0.0004	1.77 (1.29–2.50)	0.001

* Adjusted by all the demographic and clinical variables in Table 1 including age, sex, smoking, drinking, TNM stage, vessel invasion, neural invasion, lymphadenectomy

** NRG include ERCC5 rs2094258 CT/TT, re873061GA/GG, ERCC2 rs238406 TT

**Fig. 1:**
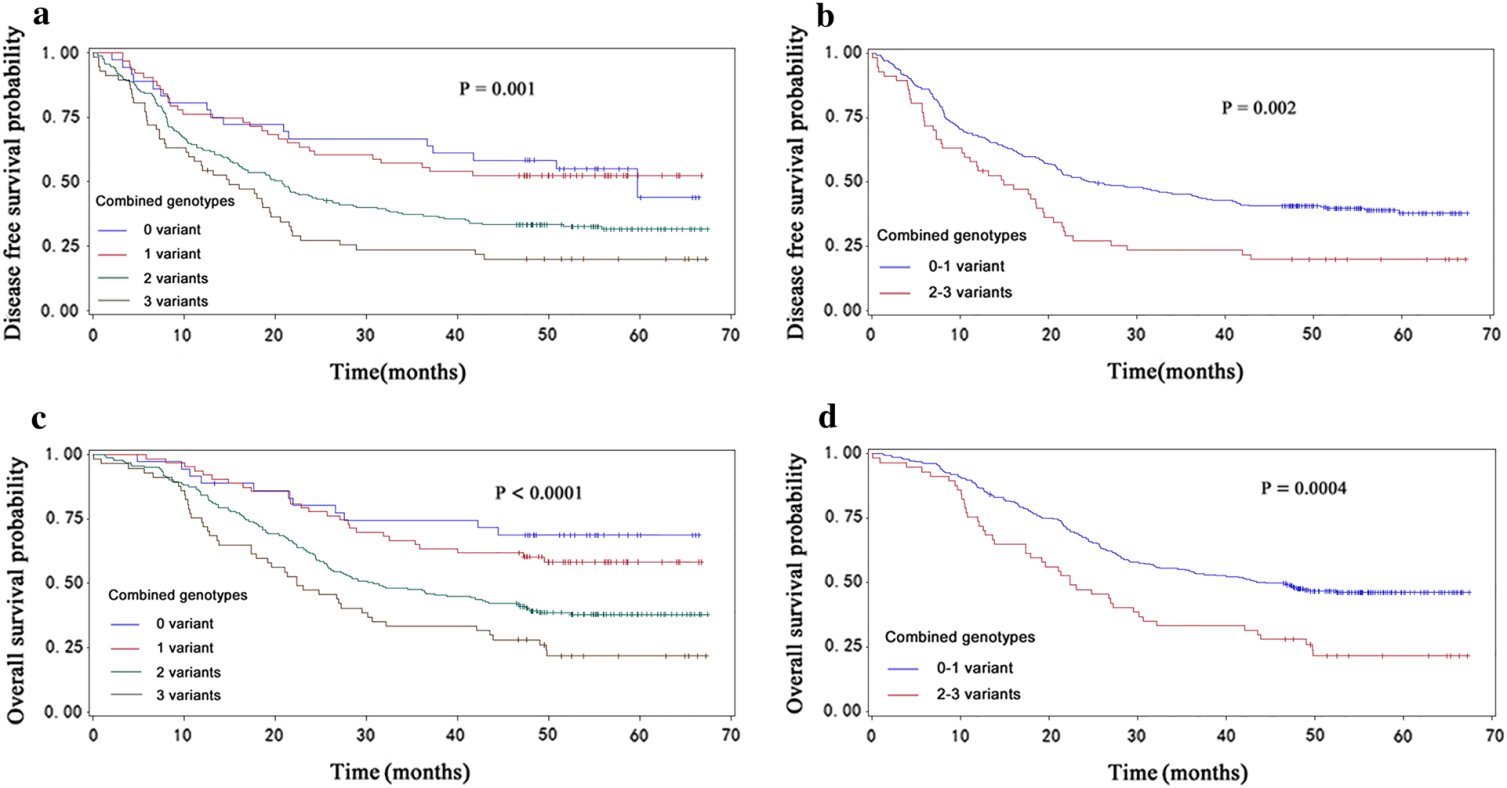
Kaplan–Meier analysis for ESCC patients by combined risk genotypes. The combined risk genotypes were composed of *ERCC5*/*XPG* rs2094258CT/TT, rs873061GA/GG and *ERCC2*/*XPG* rs238406TT). **a** DFS by 0, 1, 2 and 3 NER variant genotypes (*P* = 0.0001). **b** DFS by 0–1 and 2–3 NER variant genotypes (*P* = 0.0001). **c** OS by 0, 1 and 2 NER variant genotypes (*P* = 0.0001). **d** OS by 0–2 and 2–3 NER variant genotypes (*P* = 0.0002)

To evaluate the collective effect of the significant SNPs on patients’ DFS and OS, we combined the risk genotypes of *ERCC5*/*XPG* rs2094258CT/TT and rs873601 GA/GG and *ERCC2*/*XPD* rs238406 TT for DFS and OS into a genotype score as the number of risk genotypes (NRG). The frequencies of patients with a score of 0, 1, 2 or 3 risk genotypes were 37, 63, 187 or 57, respectively. For DFS, with the increasing NRG, patients had an increased risk of disease progression, compared with those carrying zero risk genotypes (*P*_trend_ < 0.0001) (Table 2; Fig. 1a). Similarly, with the increasing NRG, risk of death increased correspondingly (*P*_trend_ < 0.0001) (Table 2; Fig. 1c).

We then dichotomized all patients into a low-risk group (0–1 risk genotypes) (LG) and a high-risk group (2–3 risk genotypes) (HG) for further stratified analysis. Compared with the LG, the HG had an obviously reduced DFS (adjHR = 1.67, 95 % CI 1.20–2.34, and *P* = 0.003) (Table 2; Fig. 1b) and OS (adjHR = 1.77, 95 % CI 1.29–2.50, and *P* = 0.001) (Table 2; Fig. 1d).

### Stratified analysis between the risk genotypes and survival of ESCC patients

We performed stratified analysis to assess whether the combined effect of risk genotypes (HG vs. LG) on DFS and OS was modified by some important demographic and clinicopathological factors listed in Table 1. For DFS or OS, we found that ESCC patients tended to exhibit an increased risk for disease progression or death in the subgroups with younger age (<60), of male, with smoking and drinking history, with a relatively earlier stage, without vessel and neural invasion and with two-field lymphadenectomy (*P* < 0.05) (Table 3 in multivariate analysis and Additional file 2: Table S2 in univariate analysis).

**Table 3:** Stratified multivariate analysis of DFS and OS between LG* and HG* in Chinese ESCC patients

Variables	No. of patients (LG/HG)	DFS			OS		
		Progression no. (%) (LG/HG)	Multivariate analysis	*P* value	Death no. (%) (LG/HG)	Multivariate analysis	*P* value
Age							
<60	149/32	86 (57.7)/24 (75.0)	1.94 (1.21–3.10)	0.006	71 (47.7)/24 (75.0)	2.41 (1.49–3.90)	0.0004
≥60	138/25	89 (64.5)/21 (84.0)	1.40 (0.85–2.32)	0.187	81 (58.7)/20 (80.0)	1.27 (0.76–2.14)	0.360
Sex							
Male	246/49	153 (62.2)/41(83.7)	1.76 (1.24–2.51)	0.002	133 (54.1)/40 (81.6)	1.89 (1.32–2.71)	0.0005
Female	41/8	22 (53.7)/4 (50)	0.62 (0.16–2.32)	0.474	19 (46.3)/4 (50.0)	074 (0.19–2.90)	0.660
Smoking							
Never	100/22	56 (56.0)/16 (72.7)	1.54 (0.86–1.74)	0.146	45 (45.0)/16 (72.7)	1.74 (0.96–3.17)	0.069
Yes	187/35	119 (63.6)/29 (82.9)	1.71 (1.12–2.60)	0.012	107 (57.2)/28 (80.0)	1.77 (1.16–2.72)	0.008
Drinking							
No	143/28	82 (57.3)/21 (75)	1.74 (1.05–2.87)	0.032	72 (50.4)/20 (71.4)	1.87 (1.11–3.15)	0.018
Yes	144/29	93 (64.6)/24 (82.8)	1.74 (1.08–2.80)	0.022	80 (55.6)/24 (82.8)	1.93 (1.20–3.12)	0.007
Vessel invasion							
No	207/36	114 (55.1)/28 (77.8)	2.38 (1.56–3.63)	<0.001	96 (46.4)/26 (72.2)	2.07 (1.33–3.22)	0.001
Yes	80/21	61 (76.3)/17 (81.0)	1.12 (0.64–2.95)	0.699	56 (70.0)/28 (85.7)	1.61 (0.92–2.83)	0.098
Neural invasion							
No	214/40	129 (60.3)/32 (80.0)	1.72 (1.16–2.55)	0.007	111 (51.9)/30 (75.0)	1.71 (1.13–1.58)	0.011
Yes	73/17	46 (63.0)/13 (76.5)	1.29 (0.64–2.59)	0.477	41 (56.2)/14 (82.4)	1.54 (0.75–3.16)	0.241
TNM stage							
II	96/20	43 (44.8)/15 (75.0)	2.06 (1.14–3.72)	0.006	38 (39.6)/14 (70)	2.40 (1.29–4.45)	0.006
III	191/37	132 (69.1)/30 (81.1)	1.44 (0.95–2.17)	0.084	114 (59.7)/30 (81.1)	1.62 (1.07–2.45)	0.024
Lymphadenectomy							
Two fields	237/44	138 (58.2)/34 (77.3)	1.67 (1.13–2.45)	0.009	119 (50.2)/34 (77.3)	1.99 (1.34–1.94)	0.006
Three fields	50/13	37 (74.0)/11 (84.6)	2.17 (1.00–4.74)	0.051	33 (66.0)/10 (76.9)	1.59 (0.72–3.52)	0.256

* LG consisted of 0–1 risk genotypes and HG consisted of 2–3 risk genotypes

### ROC curve establish a new prognostic model with combined genotypes

**Fig. 2:**
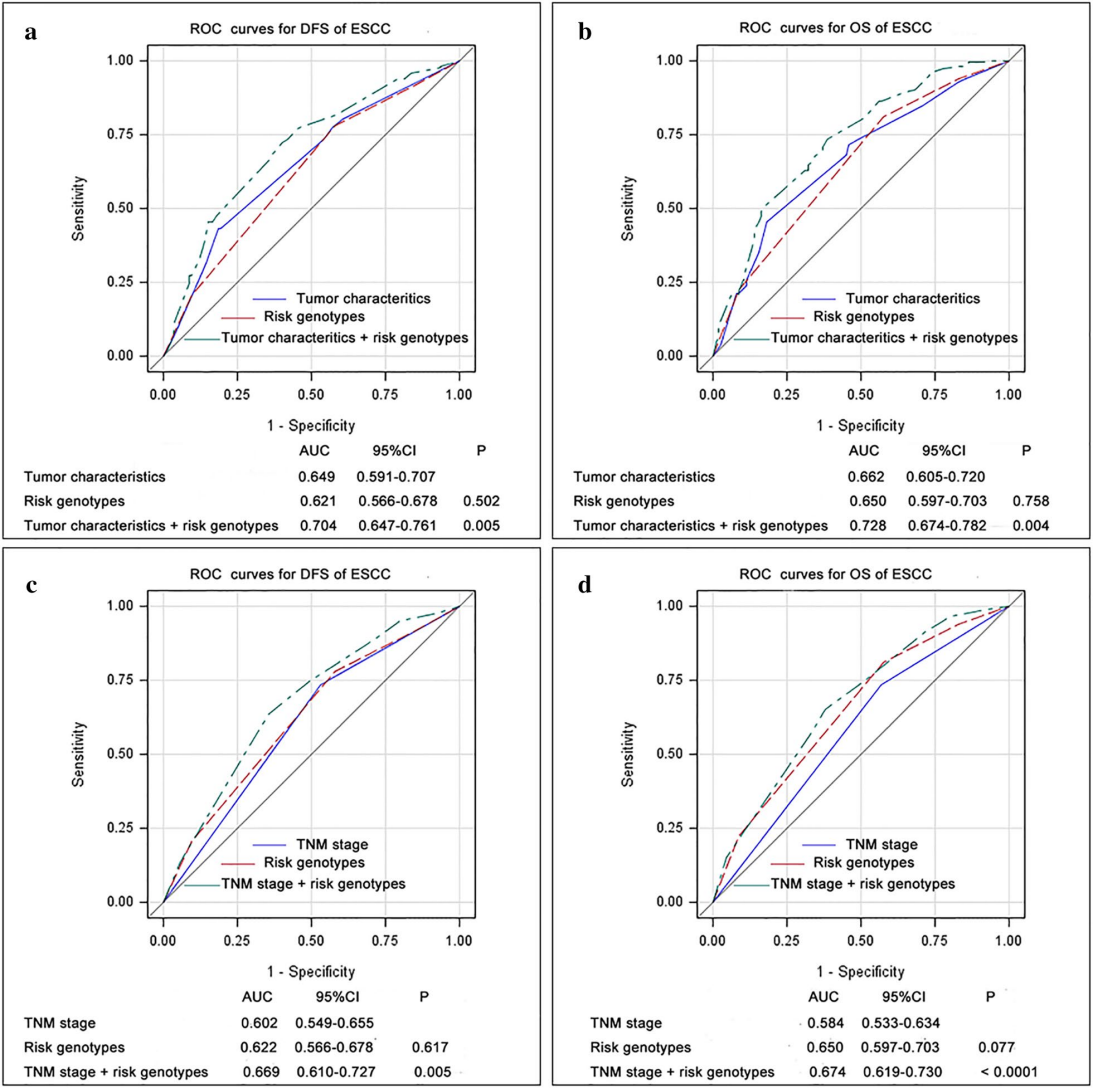
ROC analyses in ESCC patients. *P* values show the area under the ROC curves (AUC) of the three different models. Clinical characteristic include the statistically significant variables in multivariate analysis in Table 1. **a** ROC analyses of the prediction of DFS by the risk genotypes model, the clinical characteristics model, and the combined risk genotypes and clinical characteristics model. **b** ROC analyses of the prediction of DFS by the risk genotypes model, the TNM stage model, and the combined risk genotypes and TNM stage model. Clinical characteristic include the statistically significant variables in multivariate analysis in Table 1. **c** ROC analyses of the prediction of OS by the risk genotypes model, the clinical characteristics model, and the combined risk genotypes and clinical characteristics model. **d** ROC analyses of the prediction of OS by the risk genotypes model, the TNM stage model, and the combined risk genotypes and TNM stage model

Finally, we constructed a prognostic model combining all the independent prognostic factors: of risk genotypes, clinical characteristics (statistically significant factors in Table 1) and TNM stage for DFS and OS, and assessed the improvement of the model by adding risk genotypes to clinical characteristics and TNM stage by the ROC analysis. The combination of risk genotypes and clinical characteristics (AUC: 0.704, 95 % CI 0.647–0.761, *P* = 0.005 for DFS, AUC: 0.728, 95 % CI 0.674–0.782, *P* = 0.004 for OS) showed a better prognostic value than did clinical characteristics (AUC: 0.649, 95 % CI 0.591–0.707, *P* = 0.005 for DFS; AUC: 0.662, 95 % CI 0.605–0.720, *P* = 0.004 for OS) (Fig. 2a, c). Also, combination of risk genotypes and TNM stage (AUC: 0.669, 95 % CI 0.610–0.727, *P* = 0.005 for DFS, AUC: 0.674, 95 % CI 0.619–0.730, *P* < 0.0001 for OS) showed a better prognostic value than did TNM stage (AUC: 0.602, 95 % CI 0.549–0.655, *P* = 0.005 for DFS; AUC: 0.584, 95 % CI 0.533–0.634, *P* < 0.0001 for OS) (Fig. 2b, d).

## Discussion

In this study, we reported that some SNPs of the NER genes, such as *ERCC5*/*XPG* rs2094258 and rs873601 and *ERCC2*/*XPD* rs238406, may independently or jointly influence the prognosis of ESCC patients treated with PAC in eastern China. These genetic variants or genotypes, combined with some demographic and clinicopathological factors, once validated by others, may provide an improved prognostic tool for ESCC patients treated with PAC (Additional file 3: Table S3).

In the present study, we found that *ERCC5*/*XPG* rs2094258 CT/TT genotypes were associated with a decreased DFS and OS in ESCC patients treated with PAC. Although one previous study of 84 patients with squamous cell carcinomas did not find an association between the *ERCC5*/*XPG* rs2094258 SNP and response to PAC in non-small-cell lung cancer (NSCLC) [30], another study of 433 patients with advanced NSCLC did find an association between the *ERCC5*/*XPG* rs2094258 SNP and outcome of PAC, with the conclusion consistent with the present study [31]. The rs2094258 SNP is located in the 5′ UTR of *ERCC5*/*XPG*, which is a putative transcription factor binding site. Although genetic variants in gene promoters may alter gene expression levels and thus likely exert some influence on clinic outcome [25, 32, 33], there has been no report about biological or functional validation for this polymorphic site, which warrants additional mechanistic studies.

In the present study, we found that the *ERCC2*/*XPD* rs238406 TT genotype was associated with a reduced DFS and OS in ESCC patients, but few studies have reported its role in prognosis of cancer patients. One Taiwan study found that *ERCC2*/*XPD* rs238406 CC (or GG of its antisense) instead of the AA (or TT of its antisense) genotype of 185 ESCC patients with neoadjuvant chemoirradiation followed by esophagectomy could additively increase risk of death and disease progression in cisplatin-based neoadjuvant concurrent chemoradiation therapy [34]. Contrary to their results, the TT genotype was associated with the worse DFS and OS in the present study. Another study showed that rs238406 AA carriers had less efficiency of DNA adduct formation in their lymphocytes [35], suggesting that cells with the rs238406 AA genotype may have a highly-efficient capability to remove DNA adducts, leading to a relatively quicker recovery from the genotoxic effects of PAC and thus drug resistance with shortened DFS and OS. On the other hand, the rs238406 AA genotype may lead to patients’ relatively mild therapeutic toxicity. For example, one study demonstrated that patients with the rs238406 AA genotype, who received oxaliplatin-based chemotherapy, suffered less grade 3 toxicities [36]. The discrepancy between Lee’s [34] and our findings perhaps may lie in the following aspects: the present study included only patients with squamous carcinomas, while Lee’s included additional patients with adenocarcinomas; patients received chemotherapy in the present study but concurrent chemo-radiation therapy in Lee’s study; and all the patients were ethnic Han Chinese in the present study but various ethnic groups of patients in Taiwan in Lee’s study.

We also found that patients with the *ERCC5*/*XPG* rs873601 GA/GG genotypes had an increased risk of progression and death of ESCC after PAC, which was not reported before. One study reported that the rs873601 G allele was associated with better PFS and OS of patients with advanced NSCLC, which may be disease-specific and need to be validated in future studies [37].

It is likely that the effect of a single SNP on clinical outcome may be much restricted, but the combined effect of several SNPs in the same or different genes could be much greater. Indeed, we found that the collective effect of the risk genotypes identified in the present study better predicted DFS and OS of the patients. Compared with some clinical factors, genetic variants such as SNPs may have a weaker effect on prognosis. As shown by ROC curves in the present study, SNPs had almost the same effect on prognosis as the TNM stage, although it served a relatively inferior role in prognosis, compared with other clinical characteristics that included clinical characteristics such as smoking, vessel invasion, neural invasion, TNM stage and lymphadenectomy.

In the stratified analyses, we found that the genotype-survival association was more evident for a mild status of clinical characteristics, such as without vessel and neural invasion, II stage, two-field lymphadenectomy, which is consistent with what were reported in Lee’s study for EC with neoadjuvant chemoradiation and esophagectomy [34]. It is likely that the severe effects of a later TNM stage and vessel invasion as poor prognosis factors [38–40] on the survival may have masked those benefit form genetic factors.

There were some limitations in the present study. First, patients included in the analyses were from one hospital in eastern China, which may not represent the general population. Second, only six putatively functional SNPs of three NER genes were tested in the study, and there were other genetic variants in these genes or other NER pathway genes that may affect the prognosis of ESCC. Finally, the present study was retrospective instead of a prospective or randomized design, thus the bias caused by other possible factors, such as standardization of dose and the judgment of disease progress, could not have been completely excluded.

## Conclusions

In summary, we identified that *ERCC5*/*XPG* rs2094258 CT/TT and rs873601 GA/GG and *ERCC2*/*XPD* rs238406 TT genotypes may independently or jointly affect survival of ESCC patients treated with PAC. These findings, once validated in future prospective studies with large sample sizes and better study designs, will provide some promising guidance for personalized treatment for ESCC patients in the adjuvant setting in China.

## Additional files

10.1186/s12967-016-0903-z Primers used for genotyping.
10.1186/s12967-016-0903-z Stratified univariate analysis of DFS and OS between LG* and HG* in Chinese ESCC patients.
10.1186/s12967-016-0903-z The original clinical and genotype data.

## Abbreviations

adjHR: adjusted hazards ratio
AUC: area under the receiver operator characteristic curve
CI: confidence interval
DFS: disease free survival
EC: esophageal cancer
ESCC: esophageal squamous cancer cell
HG: high-risk group
LG: low-risk group
MPRCT: multicenter prospective randomized controlled trial
NER: nucleotide excision repair
NRG: number of risk genotypes
OS: overall survival
ROC: receiver operating characteristic
PAC: platinum-based adjuvant chemotherapy
SNP: single nucleotide polymorphism
UTR: untranslated region

## References

[1] Wang AH, Liu Y, Wang B, He YX, Fang YX, Yan YP (2014). Epidemiological studies of esophageal cancer in the era of genome-wide association studies. World J Gastrointest Pathophysiol.

[2] Crosby T, Evans M, Gillies RS, Maynard ND (2009). The management of a patient with an operable carcinoma of the oesophagus. Ann R Coll Surg Engl.

[3] Shapiro J, van Lanschot JJ, Hulshof MC, van Hagen P, van Berge Henegouwen MI, Wijnhoven BP, van Laarhoven HW, Nieuwenhuijzen GA, Hospers GA, Bonenkamp JJ (2015). Neoadjuvant chemoradiotherapy plus surgery versus surgery alone for oesophageal or junctional cancer (CROSS): long-term results of a randomised controlled trial. Lancet Oncol.

[4] Bosset JF, Gignoux M, Triboulet JP, Tiret E, Mantion G, Elias D, Lozach P, Ollier JC, Pavy JJ, Mercier M, Sahmoud T (1997). Chemoradiotherapy followed by surgery compared with surgery alone in squamous-cell cancer of the esophagus. N Engl J Med.

[5] Burmeister BH, Smithers BM, Gebski V, Fitzgerald L, Simes RJ, Devitt P, Ackland S, Gotley DC, Joseph D, Millar J (2005). Surgery alone versus chemoradiotherapy followed by surgery for resectable cancer of the oesophagus: a randomised controlled phase III trial. Lancet Oncol.

[6] Conroy T, Galais MP, Raoul JL, Bouche O, Gourgou-Bourgade S, Douillard JY, Etienne PL, Boige V, Martel-Lafay I, Michel P (2014). Definitive chemoradiotherapy with FOLFOX versus fluorouracil and cisplatin in patients with oesophageal cancer (PRODIGE5/ACCORD17): final results of a randomised, phase 2/3 trial. Lancet Oncol.

[7] Chiu PW, Chan AC, Leung SF, Leong HT, Kwong KH, Li MK, Au-Yeung AC, Chung SC, Ng EK (2005). Multicenter prospective randomized trial comparing standard esophagectomy with chemoradiotherapy for treatment of squamous esophageal cancer: early results from the Chinese University Research Group for Esophageal Cancer (CURE). J Gastrointest Surg.

[8] Medical Research Council Oesophageal Cancer Working, Group (2002). Surgical resection with or without preoperative chemotherapy in oesophageal cancer: a randomised controlled trial. Lancet.

[9] Allum WH, Stenning SP, Bancewicz J, Clark PI, Langley RE (2009). Long-term results of a randomized trial of surgery with or without preoperative chemotherapy in esophageal cancer. J Clin Oncol.

[10] Kelsen DP, Ginsberg R, Pajak TF, Sheahan DG, Gunderson L, Mortimer J, Estes N, Haller DG, Ajani J, Kocha W (1998). Chemotherapy followed by surgery compared with surgery alone for localized esophageal cancer. N Engl J Med.

[11] Kelsen DP, Winter KA, Gunderson LL, Mortimer J, Estes NC, Haller DG, Ajani JA, Kocha W, Minsky BD, Roth JA, Willett CG (2007). Long-term results of RTOG trial 8911 (USA Intergroup 113): a random assignment trial comparison of chemotherapy followed by surgery compared with surgery alone for esophageal cancer. J Clin Oncol.

[12] Schweigert M, Dubecz A, Stein HJ (2013). Oesophageal cancer–an overview. Nat Rev Gastroenterol Hepatol.

[13] Ando N, Iizuka T, Kakegawa T, Isono K, Watanabe H, Ide H, Tanaka O, Shinoda M, Takiyama W, Arimori M (1997). A randomized trial of surgery with and without chemotherapy for localized squamous carcinoma of the thoracic esophagus: the Japan Clinical Oncology Group Study. J Thorac Cardiovasc Surg.

[14] Ando N, Iizuka T, Ide H, Ishida K, Shinoda M, Nishimaki T, Takiyama W, Watanabe H, Isono K, Aoyama N (2003). Surgery plus chemotherapy compared with surgery alone for localized squamous cell carcinoma of the thoracic esophagus: a Japan Clinical Oncology Group Study—JCOG9204. J Clin Oncol.

[15] Lyu X, Huang J, Mao Y, Liu Y, Feng Q, Shao K, Gao S, Jiang Y, Wang J, He J (2014). Adjuvant chemotherapy after esophagectomy: is there a role in the treatment of the lymph node positive thoracic esophageal squamous cell carcinoma?. J Surg Oncol.

[16] Mathiaux J, Le Morvan V, Pulido M, Jougon J, Begueret H, Robert J (2011). Role of DNA repair gene polymorphisms in the efficiency of platinum-based adjuvant chemotherapy for non-small cell lung cancer. Mol Diagn Ther.

[17] Bosken CH, Wei Q, Amos CI, Spitz MR (2002). An analysis of DNA repair as a determinant of survival in patients with non-small-cell lung cancer. J Natl Cancer Inst.

[18] Pfeifer GP, Denissenko MF, Olivier M, Tretyakova N, Hecht SS, Hainaut P (2002). Tobacco smoke carcinogens, DNA damage and p53 mutations in smoking-associated cancers. Oncogene.

[19] Sitaram A, Plitas G, Wang W, Scicchitano DA (1997). Functional nucleotide excision repair is required for the preferential removal of N-ethylpurines from the transcribed strand of the dihydrofolate reductase gene of Chinese hamster ovary cells. Mol Cell Biol.

[20] Rosell R, Taron M, Barnadas A, Scagliotti G, Sarries C, Roig B (2003). Nucleotide excision repair pathways involved in Cisplatin resistance in non-small-cell lung cancer. Cancer Control.

[21] Gillet LC, Scharer OD (2006). Molecular mechanisms of mammalian global genome nucleotide excision repair. Chem Rev.

[22] Yokoi M, Masutani C, Maekawa T, Sugasawa K, Ohkuma Y, Hanaoka F (2000). The xeroderma pigmentosum group C protein complex XPC-HR23B plays an important role in the recruitment of transcription factor IIH to damaged DNA. J Biol Chem.

[23] Spitz MR, Wu X, Wang Y, Wang LE, Shete S, Amos CI, Guo Z, Lei L, Mohrenweiser H, Wei Q (2001). Modulation of nucleotide excision repair capacity by XPD polymorphisms in lung cancer patients. Cancer Res.

[24] Yang R, Zhang C, Malik A, Shen ZD, Hu J, Wu YH (2014). Xeroderma pigmentosum group D polymorphisms and esophageal cancer susceptibility: a meta-analysis based on case-control studies. World J Gastroenterol.

[25] Fleming ND, Agadjanian H, Nassanian H, Miller CW, Orsulic S, Karlan BY, Walsh CS (2012). Xeroderma pigmentosum complementation group C single-nucleotide polymorphisms in the nucleotide excision repair pathway correlate with prolonged progression-free survival in advanced ovarian cancer. Cancer.

[26] Li C, Yin M, Wang LE, Amos CI, Zhu D, Lee JE, Gershenwald JE, Grimm EA, Wei Q (2013). Polymorphisms of nucleotide excision repair genes predict melanoma survival. J Invest Dermatol.

[27] do Kim Y, Paek TY, Oh SY, Kim YB, Lee JH, Lee MY, Choi ZS, Suh KW (2014). Pretreatment selection of regimen according to genetic analysis improves the efficacy of chemotherapy in the first line treatment of metastatic colorectal cancer. J Surg Oncol.

[28] Liu D, Wu HZ, Zhang YN, Kang H, Sun MJ, Wang EH, Yang XL, Lian MQ, Yu ZJ, Zhao L (2012). DNA repair genes XPC, XPG polymorphisms: relation to the risk of colorectal carcinoma and therapeutic outcome with Oxaliplatin-based adjuvant chemotherapy. Mol Carcinog.

[29] DeLong ER, DeLong DM, Clarke-Pearson DL (1988). Comparing the areas under two or more correlated receiver operating characteristic curves: a nonparametric approach. Biometrics.

[30] He C, Duan Z, Li P, Xu Q, Yuan Y (2013). Role of ERCC5 promoter polymorphisms in response to platinum-based chemotherapy in patients with advanced non-small-cell lung cancer. Anticancer Drugs.

[31] Yuli Y, Zhe S, Xia W, Siqing L, Zhenxuan W, Yu-Hua Z, Bing S, Jun-Wei C (2013). XPG is a novel biomarker of clinical outcome in advanced non-small-cell lung cancer. Pak J Med Sci.

[32] Peng J, Chen YY, Yang LX, Zhao XY, Gao ZQ, Yang J, Wu WT, Wang HJ, Wang JC, Qian J (2013). XBP1 promoter polymorphism modulates platinum-based chemotherapy gastrointestinal toxicity for advanced non-small cell lung cancer patients. Lung Cancer.

[33] Guo X, Li H, Fei F, Liu B, Li X, Yang H, Chen Z, Xing J (2015). Genetic variations in SLC3A2/CD98 gene as prognosis predictors in non-small cell lung cancer. Mol Carcinog..

[34] Lee JM, Yang PW, Yang SY, Chuang TH, Tung EC, Chen JS, Huang PM, Lee YC (2011). Genetic variants in DNA repair predicts the survival of patients with esophageal cancer. Ann Surg.

[35] Zhao H, Wang LE, Li D, Chamberlain RM, Sturgis EM, Wei Q (2008). Genotypes and haplotypes of ERCC1 and ERCC2/XPD genes predict levels of benzo[a]pyrene diol epoxide-induced DNA adducts in cultured primary lymphocytes from healthy individuals: a genotype–phenotype correlation analysis. Carcinogenesis.

[36] Kweekel DM, Antonini NF, Nortier JW, Punt CJ, Gelderblom H, Guchelaar HJ (2009). Explorative study to identify novel candidate genes related to oxaliplatin efficacy and toxicity using a DNA repair array. Br J Cancer.

[37] Hu W, Pan J, Zhao P, Yang G, Yang S (2014). Genetic polymorphisms in XPG could predict clinical outcome of platinum-based chemotherapy for advanced non-small cell lung cancer. Tumour Biol.

[38] Ma G, Zhang X, Ma Q, Rong T, Long H, Lin P, Fu J, Zhang L (2015). A novel multivariate scoring system for determining the prognosis of lymph node-negative esophageal squamous cell carcinoma following surgical therapy: an observational study. Eur J Surg Oncol..

[39] Yuequan J, Shifeng C, Bing Z (2010). Prognostic factors and family history for survival of esophageal squamous cell carcinoma patients after surgery. Ann Thorac Surg.

[40] Li H, Zhang Q, Xu L, Chen Y, Wei Y, Zhou G (2009). Factors predictive of prognosis after esophagectomy for squamous cell cancer. J Thorac Cardiovasc Surg.

