# Supplementary material for: Genetic variants of DNA repair genes predict the survival of patients with esophageal squamous cell cancer receiving platinum-based adjuvant chemotherapy
Source: J Transl Med. 2016 May 31;14:154. doi: 10.1186/s12967-016-0903-z (PMC4888614; doi:10.1186/s12967-016-0903-z)
Supplement: Supplementary file 1 — 10.1186/s12967-016-0903-z Primers used for genotyping. [file 12967_2016_903_MOESM1_ESM.docx]

| **Table S1 Primers used for genotyping** | | |
| --- | --- | --- |
| **Gene** | **SNP** | **Primer sequence** |
| *ERCC5* | rs2094258 | TTATAAAGAAGTCTTTAAAAAATTG[C/T]CTGCTCCCTTAAGTCAGCCTTTTCA |
| *ERCC5* | rs2296147 | CCCGCCAACGGCCATTCTCTGGACC[C/T]GTCTTTCTTCCGGGAGGCGGTGACA |
| *ERCC5* | rs873601 | GACGCTTCGCACGGAGATTTGGGTT[C/T]CCTGGTGTTCTATTAGGCATATTTT |
| *ERCC2* | rs238406 | CCTGCCCTCCAGTAACCTCATAGAA[G/T]CGGCAGTGGGGCAGGCTGGTGTCAT |
| *XPC* | rs1870134 | TTGGCCTTGGATTTCTGGCTGCGCA[C/G]TTCGCGTCCCCGCGGCTCCCCGCCG |
| *XPC* | rs2228001 | TCTAGTGGGCGCTCAGCTCACAGCT[G/T]CTCAAATGGGAACAGGTGGGAAGCT |
