# Supplementary material for: Genetic variants of DNA repair genes predict the survival of patients with esophageal squamous cell cancer receiving platinum-based adjuvant chemotherapy
Source: J Transl Med. 2016 May 31;14:154. doi: 10.1186/s12967-016-0903-z (PMC4888614; doi:10.1186/s12967-016-0903-z)
Supplement: Supplementary file 2 — 10.1186/s12967-016-0903-z Stratified univariate analysis of DFS and OS between LG* and HG* in Chinese ESCC patients. [file 12967_2016_903_MOESM2_ESM.docx]

| **Table S2 Stratified univariate analysis of DFS and OS between LG* and HG* in Chinese ESCC patients** | | | | | | | | | |
| --- | --- | --- | --- | --- | --- | --- | --- | --- | --- |
| **Variable** | **No. of Patients (LG/HG)** |  | **DFS** | | |  | **OS** | | |
|  |  |  | **Progression**  **No. (%) (LG/HG)** | **Univariate analysis** | ***P* value** |  | **Death**  **No. (%) (LG/HG)** | **Univariate analysis** | ***P* value** |
| Age |  |  |  |  |  |  |  |  |  |
| < 60 | 149/32 |  | 86 (57.7)/24 (75.0) | **1.92 (1.22-3.02)** | **0.006** |  | 71 (47.7)/24 (75.0) | **2.37 (1.49-3.76)** | **0.0003** |
| ≥ 60 | 138/25 |  | 89 (64.5)/21 (84.0) | 1.47 (0.91-2.37) | 0.187 |  | 81 (58.7)/20 (80.0) | 1.37 (0.83-2.26) | 0.133 |
| Sex |  |  |  |  |  |  |  |  |  |
| Male | 246/49 |  | 153 (62.2)/41(83.7) | **1.90 (1.34-2.69)** | **0.002** |  | 133 (54.1)/40 (81.6) | **2.01 (1.19-2.87)** | **0.0001** |
| Female | 41/8 |  | 22 (53.7)/4 (50) | 0.85 (0.29-2.46) | 0.474 |  | 19 (46.3)/4 (50.0) | 1.00 (0.34-2.93) | 0.993 |
| Smoking |  |  |  |  |  |  |  |  |  |
| Never | 100/22 |  | 56 (56.0)/16 (72.7) | 1.51 (0.87-1.64) | 0.146 |  | 45 (45.0)/16 (72.7) | 1.74 (0.98-3.08) | 0.060 |
| Yes | 187/35 |  | 119 (63.6)/29 (82.9) | **1.87 (1.24-2.82)** | **0.012** |  | 107 (57.2)/28 (80.0) | **2.00 (1.32-3.03)** | **0.001** |
| Drinking |  |  |  |  |  |  |  |  |  |
| No | 143/28 |  | 82 (57.3)/21 (75) | **1.53 (1.01-2.48)** | **0.032** |  | 72 (50.4)/20 (71.4) | **1.67 (1.01-2.75)** | **0.043** |
| Yes | 144/29 |  | 93 (64.6)/24 (82.8) | **1.89 (1.20-2.97)** | **0.022** |  | 80 (55.6)/24 (82.8) | **1.99 (1.26-3.15)** | **0.003** |
| Vessel invasion |  |  |  |  |  |  |  |  |  |
| No | 207/36 |  | 114 (55.1)/28 (77.8) | **2.02 (1.34-3.07)** | **< 0.001** |  | 96 (46.4)/26 (72.2) | **1.97 (1.28-3.05)** | **0.002** |
| Yes | 80/21 |  | 61 (76.3)/17 (81.0) | 1.09 (0.64-2.87) | 0.699 |  | 56 (70.0)/28 (85.7) | 1.47 (0.86-2.50) | 0.158 |
| Neural invasion |  |  |  |  |  |  |  |  |  |
| No | 214/40 |  | 129 (60.3)/32 (80.0) | **1.73 (1.18-2.56)** | **0.007** |  | 111 (51.9)/30 (75.0) | **1.78 (1.19-2.67)** | **0.005** |
| Yes | 73/17 |  | 46 (63.0)/13 (76.5) | 1.59 (0.86-2.96) | 0.477 |  | 41 (56.2)/14 (82.4) | 1.95 (1.06-3.59) | 0.031 |
| TNM Stage |  |  |  |  |  |  |  |  |  |
| II | 96/20 |  | 43 (44.8)/15 (75.0) | **2.40 (1.29-4.44)** | **0.006** |  | 38 (39.6)/14 (70) | **2.17 (1.17-4.01)** | **0.013** |
| III | 191/37 |  | 132 (69.1)/30 (81.1) | **1.60 (1.07-2.38)** | **0.084** |  | 114 (59.7)/30 (81.1) | **1.77 (1.18-2.65)** | **0.006** |
| Lymphadenectomy |  |  |  |  |  |  |  |  |  |
| Two fields | 237/44 |  | 138 (58.2)/34 (77.3) | **1.69 (1.16-2.46)** | **0.009** |  | 119 (50.2)/34 (77.3) | **1.92 (1.31-1.82)** | **0.008** |
| Three fields | 50/13 |  | 37 (74.0)/11 (84.6) | 1.60 (0.82-3.14) | 0.051 |  | 33 (66.0)/10 (76.9) | 1.46 (0.72-2.97) | 0.296 |
| *LG consisted of 0-1 risk genotype and HG consisted of 2-3 risk genotypes. | | | | | | | | | |
